# Towards mechanobiologically optimized mandible reconstruction: CAD/CAM miniplates vs. reconstruction plates for fibula free flap fixation: A finite element study

**DOI:** 10.3389/fbioe.2022.1005022

**Published:** 2022-11-17

**Authors:** Philipp Ruf, Vincenzo Orassi, Heilwig Fischer, Claudius Steffen, Georg N. Duda, Max Heiland, Kilian Kreutzer, Sara Checa, Carsten Rendenbach

**Affiliations:** ^1^ Department of Oral and Maxillofacial Surgery, Charité—Universitätsmedizin, Corporate member of Freie Universität Berlin, Humboldt-Universität zu Berlin and Berlin Institute of Health, Berlin, Germany; ^2^ Julius Wolff Institute, Berlin Institute of Health at Charité—Universitätsmedizin Berlin, Berlin, Germany

**Keywords:** mandibular reconstruction, fibula free flap, CAD/CAM, miniplate, reconstruction plate, finite element, mechanobiology

## Abstract

Due to their advantages in applicability, patient-specific (CAD/CAM) reconstruction plates are increasingly used in fibula free flap mandible reconstruction. In addition, recently, CAD/CAM miniplates, with further advantages in postoperative management, have been introduced. However, biomechanical conditions induced by CAD/CAM systems remain partially unknown. This study aimed to evaluate the primary fixation stability of CAD/CAM fixators. For a patient-specific scenario, the biomechanical conditions induced in a one segmental fibula free flap stabilized using either a CAD/CAM reconstruction plate or CAD/CAM miniplates were determined using finite element analysis. The main output parameters were the strains between intersegmental bone surfaces and stresses in the fixation systems due to different biting scenarios. CAD/CAM miniplates resulted in higher mechanical strains in the mesial interosseous gap, whereas CAD/CAM reconstruction plate fixation resulted in higher strains in the distal interosseous gap. For all investigated fixation systems, stresses in the fixation systems were below the material yield stress and thus material failure would not be expected. While the use of CAD/CAM miniplates resulted in strain values considered adequate to promote bone healing in the mesial interosseous gap, in the distal interosseous gap CAD/CAM reconstruction plate fixation might result in more beneficial tissue straining. A mechanical failure of the fixation systems would not be expected.

## 1 Introduction

Mandibular reconstruction after segmental resection due to tumor or osteonecrosis is usually performed with myoosseous or osteomyocutaneous free flaps (AO Surgery Reference, 2013a). Due to its bone anatomical shape and length, long vascular pedicle and the possibility to, both, include up to two skin islands and to work in two parallel teams at the neck and leg intraoperatively, the fibula free flap is prioritized by most maxillofacial surgeons (Brown et al., 2017; Rendenbach et al., 2018). The gold standard in fibula free flap fixation are conventional, intraoperatively hand-bent reconstruction plates (Rendenbach et al., 2018). From a biomechanical perspective, these bicortical fixed and thick plates bear loads to provide maximum primary fixation stability (Wądołowski et al., 2020; AO Surgery Reference, 2021b). Alternatively, smaller and monocortically fixed conventional miniplates are intraoperatively bent and used for fibula free fixation to share the load with the stabilized bone (Champy et al., 1978; Rendenbach et al., 2017; Wądołowski et al., 2020; AO Surgery Reference, 2021b). However, no superiority in functional outcomes has been proven comparing these conventional systems (Schupp et al., 2007; Robey et al., 2008; Fontana et al., 2016; Steffen et al., 2020).

Over the last years, there has been an increase in the use of virtual surgical planning and computer-aided design/computer-aided manufacturing (CAD/CAM) in maxillofacial surgery (Rendenbach et al., 2018). This approach provides high accuracy and easier application than the conventional hand-bent fixation systems (Rendenbach et al., 2018). However, CAD/CAM reconstruction plates provide an even higher stiffness than conventional plates, therefore could decrease the intersegmental strains and negatively influence bone healing (Rendenbach et al., 2017; Rendenbach et al., 2019). To prevent this effect and to improve soft tissue management and dental rehabilitation, while using the advantages of CAD/CAM systems, the use of CAD/CAM miniplates has recently been proposed (Kreutzer et al., 2022a; Kreutzer et al., 2022b). However, to our knowledge, it remains unknown whether the biomechanical conditions provided by CAD/CAM miniplates are more favorable compared to the ones provided by CAD/CAM reconstruction plates. Since primary fixation stability is known to crucially affect the bone healing outcome (Claes and Heigele, 1999), the biomechanical conditions induced by the different CAD/CAM fixation systems within the intersegmental healing regions need to be investigated.

This study aimed to biomechanically compare load-bearing titanium CAD/CAM reconstruction plates and load-sharing titanium CAD/CAM miniplates. To this aim, finite element (FE) analyses were performed to quantify the biomechanical conditions using such fixation strategies in reconstructed mandibles. We analyzed the mechanical tissue straining of the healing region as well as the mechanical stresses in the fixation systems for CAD/CAM reconstruction plates and CAD/CAM miniplates.

## 2 Materials and methods

Three-dimensional (3D) FE models of a reconstructed mandible with a fibular segment, stabilized with CAD/CAM miniplates or reconstruction plates, were developed.

### 2.1 Model geometry

**FIGURE 1:**
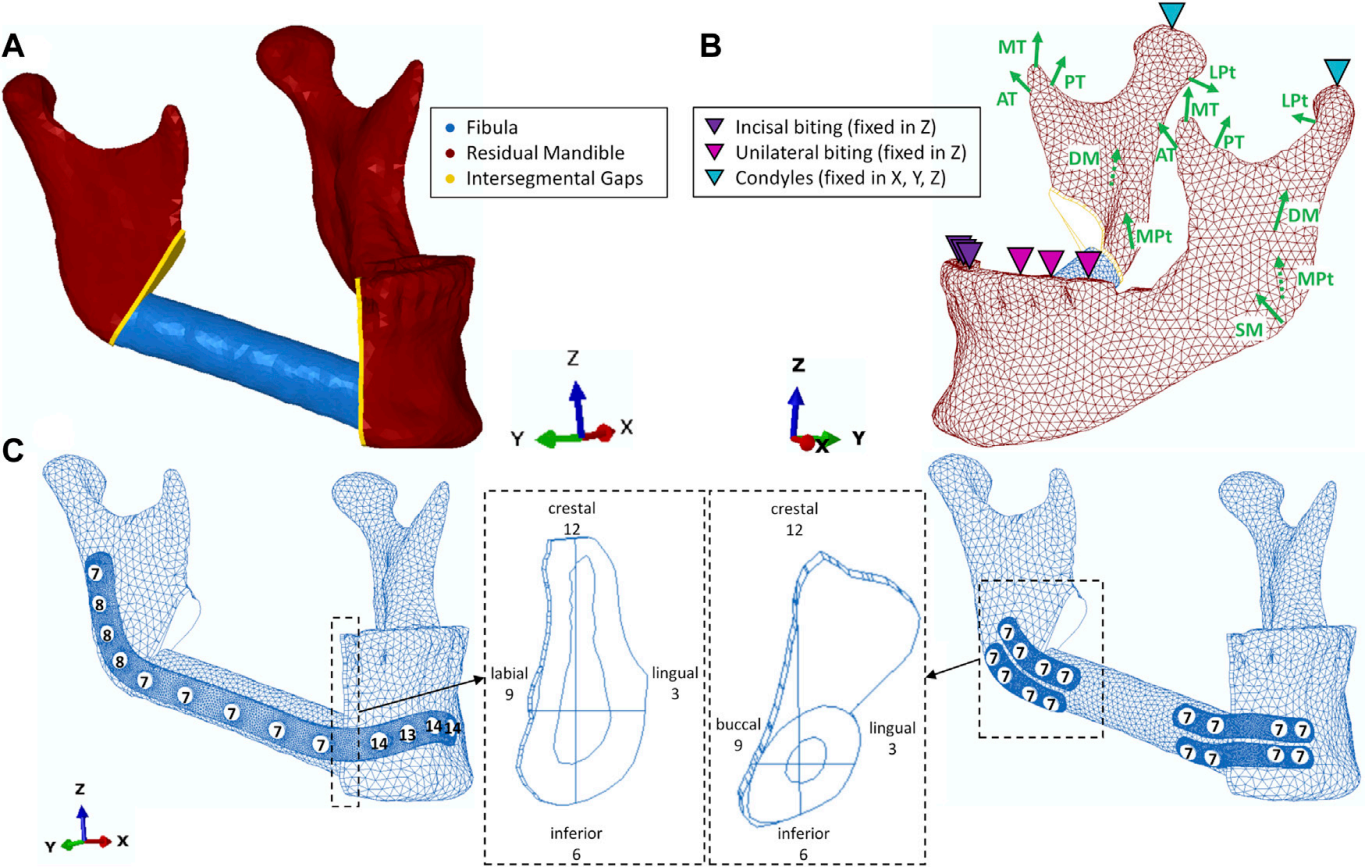
**(A)** geometry without fixation; **(B)** loading and boundary conditions including muscles superficial- (SM) and deep (DM) masseter, medial- (MPt) and lateral (LPt) pterygoid, anterior- (AT), medial- (MT) and posterior (PT) temporalis; **(C)** reconstruction plate and miniplate with screws and screw lengths (in mm), intersegmental gaps with anatomical and given clock directions.

FE models were developed based on the pre-operative computed tomography (CT) imaging data of a patient who underwent a segmental mandible resection from the mandibular angle until the right canine region. The patient was a 57-year-old woman suffering from oral squamous cell carcinoma of the right mouth floor with bone invasion. The CT scan was performed using an axial mode. The voxel size was 0.5 mm × 0.5 mm, and the slice thickness was 0.625 mm (General Electric, Boston, Massachusetts, United States). The resulting DICOM images were imported into commercial segmentation and meshing Software Amira 6.0.1 (ZUSE Institute, Berlin, Germany). After segmentation, the mandible and right fibula were virtually differentiated into cortical and trabecular bone based on their greyscale values. Cortical bone was initially segmented using Hounsfield Unit ranges from 250 to 2,200 for the fibula and 300-2,200 for the mandible. Subsequently, the remaining inner areas of the bones were defined as trabecular bone. Thereafter, a primary mesh was assigned to the segmented bones. The bones were exported as a .stl file into the 3D Computer Aided Design (CAD) software SolidWorks 2020 (Dassault Systèmes Vélizy-Villacoublay, France) to perform the virtual resection of the mandible and the placement of the fibula flap. Virtual resection was conducted on the right side of the mandible going from the mandibular angle to the right canine using a help geometry in form of an extruded trapeze. To fill the gap, a complementary autologous fibula flap was virtually harvested from the distal right fibula according to recent surgery guidelines (AO Surgery Reference, 2013a), where an 8 cm residual stump length of the fibula remained. The resulting fibula segment had an average length of 5 cm and a maximum diameter of 1.2 cm. Residual mandible cortical and trabecular geometry, along with the complementary fibula cortical and trabecular geometry and their meshes were imported as volume parts into the FEA software Abaqus CAE 2019 (Dassault Systèmes Vélizy-Villacoublay, France), where they were merged into one volume part with four subdivided geometry sets. In this part, the intersegmental gaps were defined by creating a linear extrude of all participating bony structures with a width of 1 mm. As a result, the residual mandible, complementary fibula, and intersegmental gaps form one continuous body with locally varying materials. 1mm as width of the intersegmental gaps was chosen since recent findings show a negative impact of excessive gap width on the bone healing outcome and 1 mm as optimal width (Steffen et al., 2022). The intersegmental gaps represent the regions of interest (ROIs) where mechanical strains are evaluated (Figure 1).

### 2.2 Fixation

CAD/CAM miniplates and reconstruction plates were designed by an experienced engineer in collaboration with the senior author to follow in-house and regulatory standards in patient-specific plate creation (Karl Leibinger Medizintechnik GmbH & Co. KG, Tuttlingen, Germany). Plates were designed using the software Geomagic Freeform Plus v2021.0.56 (3D Systems Rock Hill, United States of America). One reconstruction plate with a thickness of 2.0 mm and 13 holes (4 distal and mesial in the mandible, 5 in the fibula) was created as well as 4 miniplates with a thickness of 1.0 mm and 4 holes each, according to load-sharing principles (AO Surgery Reference, 2021a). The plates were placed using the software SolidWorks 2020 and fitting screws were designed using a simplified geometry (no threats) to minimize computational costs.

To achieve load-sharing osteosynthesis of the underlying bony tissues, 7 mm monocortical screws were used to fix the miniplates (Steffen et al., 2020). Screws of 2 mm diameter and 7 mm length were chosen for the fibula in all fixation scenarios to prevent damage to the intraosseous vessels (AO Surgery Reference, 2013a). For the reconstruction plate, load-bearing principles were applied to provide absolute stability. Therefore, bicortical screws of 2 mm diameter and for each location defined length according to the CT scan were placed on the mandible (AO Surgery Reference, 2021b). Subsequently, the geometry of all fixation devices was exported as volume parts from Solidworks 2020 to Abaqus CAE 2019.

### 2.3 Meshing and mesh convergency study

All geometries were meshed in Abaqus CAE 2019 using quadratic tetrahedral (second order) elements (Type C3D10). For the fixation devices, this resulted in an average element size of 0.5 mm on the reconstruction plate (RP), 0.33 mm in the miniplates (MPs) and 1 mm in the screws.

For the ROIs (intersegmental gaps), a mesh convergency study was performed with four different mesh sizes. To reduce the computational costs of the mesh convergency analysis, the biomechanical behavior of the reconstructed mandible (continuous body of residual mandible, complementary fibula, and intersegmental gaps) was analyzed without a fixation device. In addition, only two force vectors were defined, inserting at both lateral angles at the insertion area of the superficial masseter (Fx = 0 N, Fy = -5 N, Fz = 5 N; Figure 1), which represents the most important chewing muscle. The finest mesh (A) resulted in an average element size of 0.15 mm in the intersegmental gaps. The increasingly coarse meshes, with average element sizes of 0.2 (B), 0.3 (C), and 0.35 mm (D), were all compared to the next finest mesh. The condyles were locked in all degrees of freedom and unilateral biting was simulated by restricting vertical displacement of the molars and premolars on the defect’s contralateral side. In both ROIs, the averages were calculated for maximum principal strain, minimum principal strain, and von Mises stress with each mesh. Subsequently, the mean values were compared for the different meshes. For both ROIs, mesh B was considered a good trade-off between accuracy and computational costs (relative error <5% for both gaps) and was therefore chosen for further analyses.

### 2.4 Constraints, loading, and boundary conditions

To simulate locking screws, regularly used in fibula free flap fixation (AO Surgery Reference, 2013b; Kreutzer et al., 2022b), tie constraints were defined between screws and plates as well as between screws and underlying bone tissue. For both fixation systems, unilateral (UNI) and incisal (INC) biting were simulated as biting tasks. Occlusion was simulated by not allowing vertical displacement for all incisors (INC) and the premolars and first molar (UNI) on the defect’s contralateral side (left) in the same setting as in previous studies (Korioth and Hannam, 1994; Orassi et al., 2021). These biting tasks were chosen to simulate a postoperative scenario according to studies evaluating the functional outcome of fibula free flap (Sakuraba et al., 2017). The condyles (COND) were assumed locked in the glenoid fossa for both biting tasks and thus they were restrained from movement in all six degrees of freedom (Figure 1).

The main participating muscles were simulated, including superficial (SM) and deep (DM) masseter; anterior (AT), medial (MT), and posterior (PT) temporalis, medial pterygoid (MPt), and inferior lateral pterygoid (LPt). Maximum muscle forces and fiber activations were obtained from previous studies (Korioth et al., 1992; Korioth and Hannam, 1994) and were modified to simulate a postoperative scenario. Therefore, on the defect’s ipsilateral side, the superficial masseter was detached and the deep masseter’s partial detachment was simulated using a 50% reduced fiber activation. A reduction of total maximum muscle forces to 12.5% was assumed. This resulted in a biting force of 45 N which is in agreement with reported values post-surgery (Gheibollahi et al., 2021). Maximum muscle forces in the healthy and postoperative scenario, fiber directions, and fiber activation are shown in Table 1. The forces are applied in relation to the frontal (XY), transversal (XZ), and sagittal (YZ) planes (Korioth and Hannam, 1994). All loading and boundary conditions are applied to one reference point and spatially distributed over a defined area to avoid stress concentrations.

**TABLE 1:** Maximum Muscle Forces, fiber direction, and fiber activation in the biting tasks unilateral (UNI) and incisal (INC) biting.

			Fiber direction				Fiber activation			
	Maximum muscle force (N)	12.5% of maximum muscle force (N)	X	X	Y	Z	INC	INC	UNI	UNI
			Right	Left			Right	Left	Right	Left
Superficial masseter	190.4	23.8	−0.207	0.207	0.884	0.419	0	0.4	0	0.72
Deep masseter	81.6	10.2	−0.546	0.546	0.758	−0.358	0.13	0.26	0.3	0.72
Medial pterygoid	174.8	21.85	0.486	−0.486	0.791	0.373	0.78	0.78	0.6	0.84
Lateral pterygoid	66.9	8.3625	0.63	−0.63	−0.174	0.757	0.71	0.71	0.65	0.3
Anterior temporalis	158	19.75	−0.149	0.149	0.988	0.044	0.08	0.08	0.58	0.73
Middle temporalis	95.6	11.95	−0.222	0.222	0.837	−0.5	0.06	0.06	0.67	0.66
Posterior temporalis	75.6	9.45	−0.208	0.208	0.474	−0.855	0.04	0.04	0.39	0.59

#### 2.5 Material properties

Cortical bone of fibula and mandible was considered as anisotropic, homogeneous, and linear elastic. The anisotropic material properties for the cortical mandible areas symphysis, body, angle, ramus, condyle, and coronoid are based on previous studies (Schwartz-Dabney and Dechow, 2003; Lovald et al., 2009). For the cortical fibula, anisotropic Young’s moduli and shear moduli are based on Lefèvre et al. (2015), while the Poisson’s ratios were taken from analogous tibia areas (Rho, 1996). The anisotropic material properties were assigned in local coordinate systems. All other materials were considered isotropic, homogeneous, and linear elastic. Values for Young’s moduli and Poisson’s ratios of the mandible’s trabecular bone (Lakatos et al., 2014), dentine (Korioth and Hannam, 1994), granulation tissue in the initial healing phase (Leong and Morgan, 2008), and the fibula’s trabecular bone (Sudneva et al., 2020) were obtained from the literature. Granulation tissue material properties were assigned to the intersegmental gaps to reproduce the initial healing phase after mandibular reconstruction. Titanium alloy material properties were assigned to plates and screws. Titanium’s yield strength was considered equal to 830 MPa (Matweb, LLC, 2022) and was used as an indicator for material failure. All material properties are listed in Table 2.

**TABLE 2:** Anisotropic and isotropic material properties for bony tissues, interosseous volume, and fixation devices. Direction 1: longitudinal or axial; Direction 2: tangential; Direction 3: transverse.

Material	Symphysis	Body	Angle	Ramus	Condyle	Coronoid	Fibula cortical	Dentin	Mandible trabecular	Fibula trabecular	Granulation tissue	Ti-6AI-4V
E1 (MPa)	20,492	21,728	23,793	24,607	23,500	28,000	28,000	17,600	300	292	1	114,000
E2 (MPa)	16,350	17,828	19,014	18,357	17,850	17,500	17,700	17,600	300	292	1	114,000
E3 (MPa)	12,092	12,700	12,757	12,971	12,650	14,000	17,700	17,600	300	292	1	114,000
Nu12	0.34	0.34	0.3	0.28	0.24	0.23	0.237	0.34	0.3	0.3	0.3	0.33
Nu23	0.22	0.2	0.22	0.23	0.25	0.28	0.231	0.34	0.3	0.3	0.3	0.33
Nu13	0.43	0.45	0.41	0.38	0.32	0.28	0.42	0.34	0.3	0.3	0.3	0.33
G12 (MPa)	6,908	7,450	7,579	7,407	7,150	7,150	4,690	6,567	115.4	112.3	0.385	44,000
G23 (MPa)	4,825	5,083	4,986	5,014	5,150	5,300	3,600	6,567	115.4	112.3	0.385	44,000
G13 (MPa)	5,317	5,533	5,493	5,386	5,500	5,750	4,720	6,567	115.4	112.3	0.385	44,000

### 2.6 Output evaluation

The intersegmental gaps as ROIs were differentiated into quadrants, using the sagittal and transversal planes to provide a high resolution of biomechanical output parameters in the ROIs. The quadrants were assigned time values (12-3, 3-6, etc.) from the anterior perspective (Figure 1)

In the ROIs, the average of the maximum and minimum principal strains was calculated for every quadrant. In the calculation, only absolute strain values above 500 µ were included. This filter was applied to minimize the influence of elements that are not part of the interosseous interface and thus, have very small strain values. By applying this operation, different sized quadrants can be compared since only regions under relatively large strains are included in the averaging.

Von Mises stress in comparison to the fixation material’s yield stress was used as a predictor for material failure. Maximum von Mises stress was calculated by averaging the 10 highest values in the percentile group [0%; 99.99%]. The largest 0.01% of stress values were excluded to minimize the effect of stress singularities that could occur by tie constraints between objects with different mesh sizes.

All values were collected from the integration point of each element to avoid possible discontinuities of the strain field at the element edges (Orassi et al., 2022).

## 3 Results

The calculated bite force values are 44.5 N for reconstruction plate and 45 N for miniplates in unilateral biting and 11.5 N for reconstruction plate and 12 N for miniplates in incisal biting.

**FIGURE 2:**
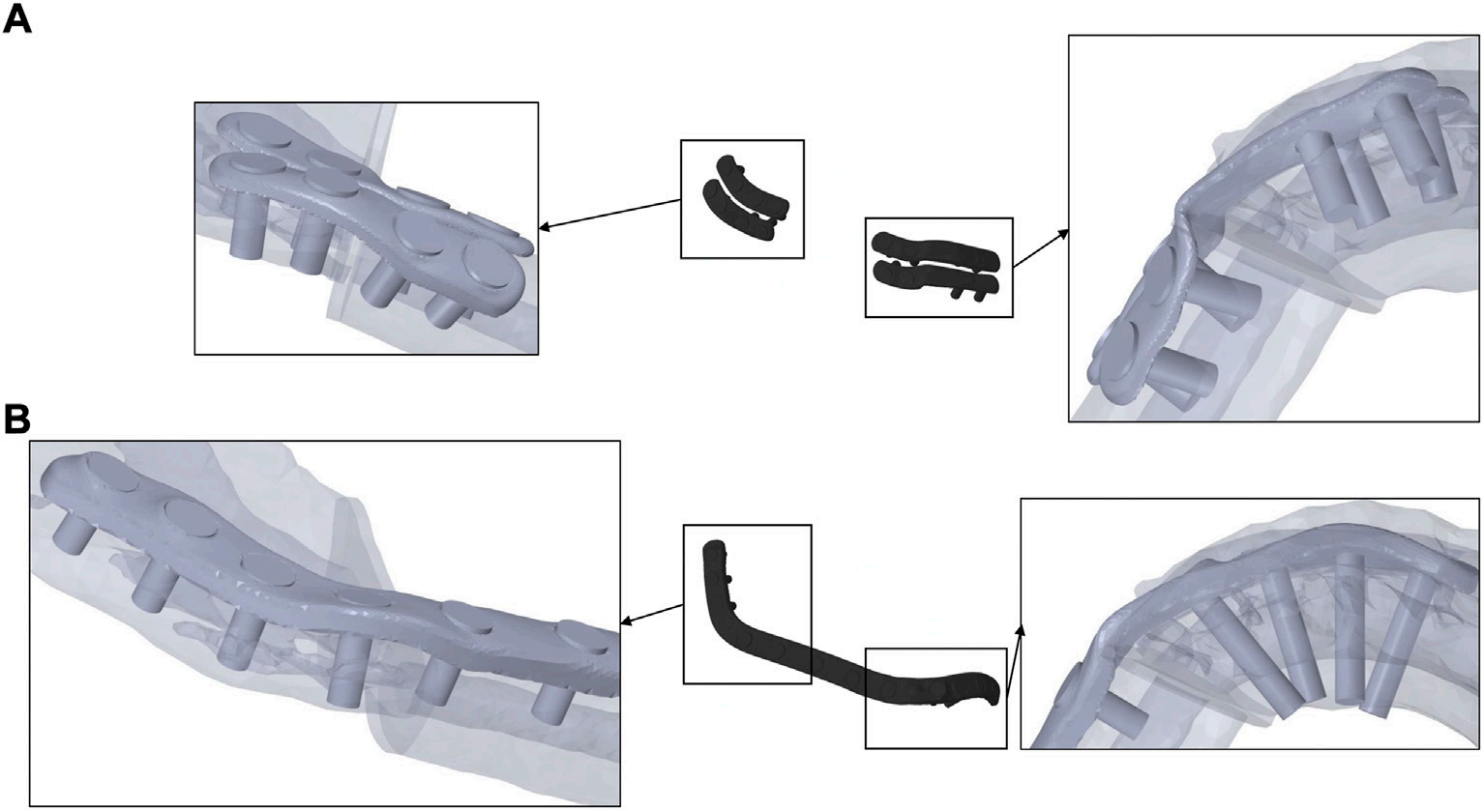
Miniplates **(A)**, fixed monocortically in the mesial and bicortically in the distal healing site, and reconstruction plate **(B)**, fixed bicortically in both healing sites, on transparent mandible from a caudal perspective.

Figure 2 shows the different fixators in comparison to the investigated patient’s bone anatomy. Monocortical fixation, using 7 mm screws, could be achieved for miniplate fixation in the mesial healing region. In the distal healing region, 7 mm screws resulted in bicortical fixation due to anatomically thin bone. For the reconstruction plate, in both healing regions, bicortical fixation of the mandible could be achieved.

### 3.1 CAD/CAM reconstruction plate fixation leads to increased strains in the distal intersegmental gap, whereas miniplate fixation increases the strains in the mesial healing site

**FIGURE 3:**
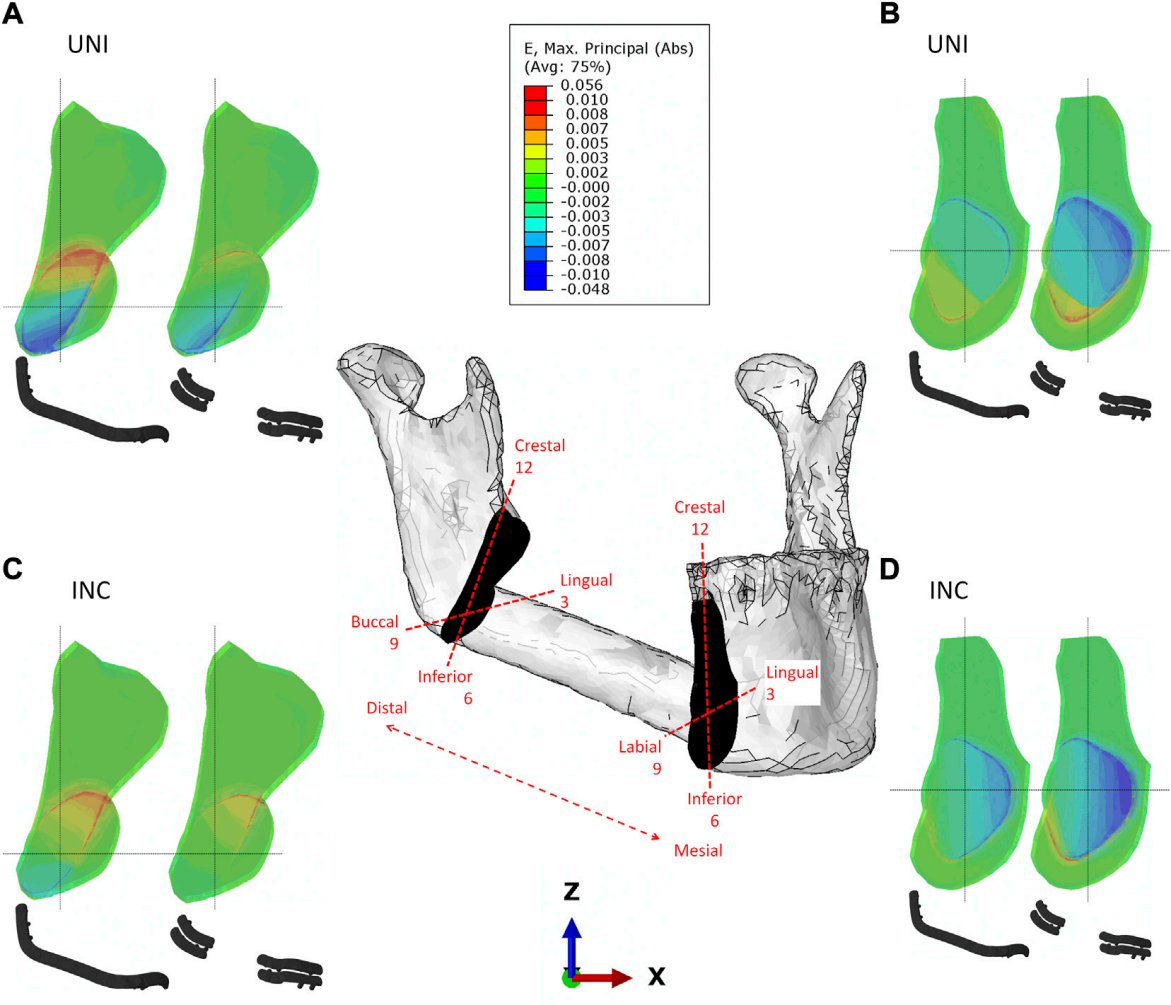
Strain distribution in the distal **(A–C)** and mesial **(B–D)** intersegmental gap for reconstruction plate and miniplate fixation in biting tasks unilateral (UNI) and incisal (INC) biting.

In the distal site, in all quadrants, average mechanical strains induced within the healing region with miniplates (range 0.07%–0.36%) were smaller than those induced with reconstruction plates (range 0.08%–0.54%) (Figure 3). However, in the mesial site, in all quadrants, higher average mechanical strains in the healing region were determined with miniplates (range 0.14%–0.61%) compared with reconstruction plate (range 0.1%–0.41%) fixation (Figure 3).

In the distal intersegmental gap, tension was determined in the crestal areas while compressive strains were determined in the inferior areas (Figure 3). Within the mesial healing region, compression is dominant lingually, while tension can be noted in the inferior, buccal regions (Figure 3).

When comparing the different biting tasks, unilateral biting results in higher strains in the distal gap, whereas incisal biting results in higher strains mesially. Quantitative values for the average maximum and minimum principal strains induced by different biting tasks and fixation systems can be found in Table 3.

**TABLE 3:** Averages of Maximum (MAX) and Minimum (MIN) principal strains for reconstruction plate (RP) and miniplate (MP) fixation in biting tasks unilateral (UNI) and incisal (INC) biting across clock-oriented quadrants in the mesial (M) and distal (D) healing site. M-12-3, M-3-6, M-6-9, and M-9-12 are the four quadrants in the mesial side with a clockwise annotation. D-12-3, D-3-6, D-6-9, and D-9-12 are the four quadrants in the distal side with a clockwise annotation. All values are given in microstrain.

Quadrants	**M**-**12**-**3**	**M**-**3**-**6**	**M**-**6**-**9**	**M**-**9**-**12**	**D**-**12**-**3**	**D**-**3**-**6**	**D**-**6**-**9**	**D**-**9**-**12**
UNI-MAX-RP	1,134	1,542	1,835	1,492	4,058	1,196	1,244	3,560
UNI-MAX-MP	2,096	2,364	2,636	1,774	1,824	871	863	1,460
INC-MAX-RP	1,484	1,107	1,303	1,033	3,456	2,025	1,605	2,426
INC-MAX-MP	1,489	1,395	2,091	1,764	2,789	1,214	857	1,568

Quadrants	**M**-**12**-**3**	**M**-**3**-**6**	**M**-**6**-**9**	**M**-**9**-**12**	**D**-**12**-**3**	**D**-**3**-**6**	**D**-**6**-**9**	**D**-**9**-**12**
UNI-MIN-RP	−2,457	−2,356	−1,834	−1,978	−4,068	−4,319	−5,413	−2,842
UNI-MIN-MP	−4,643	−5,492	−3,821	−3,960	−1,969	−3,517	−3,577	−1,441
INC-MIN-RP	−3,871	−4,149	−2,290	−2,053	−4,311	−1,425	−2,204	−849
INC-MIN-MP	−5,090	−6,112	−3,873	−2,830	−1,099	−675	−942	−826

### 3.2 Mechanical stresses within CAD/CAM reconstruction and miniplates remain below failure ranges

**FIGURE 4:**
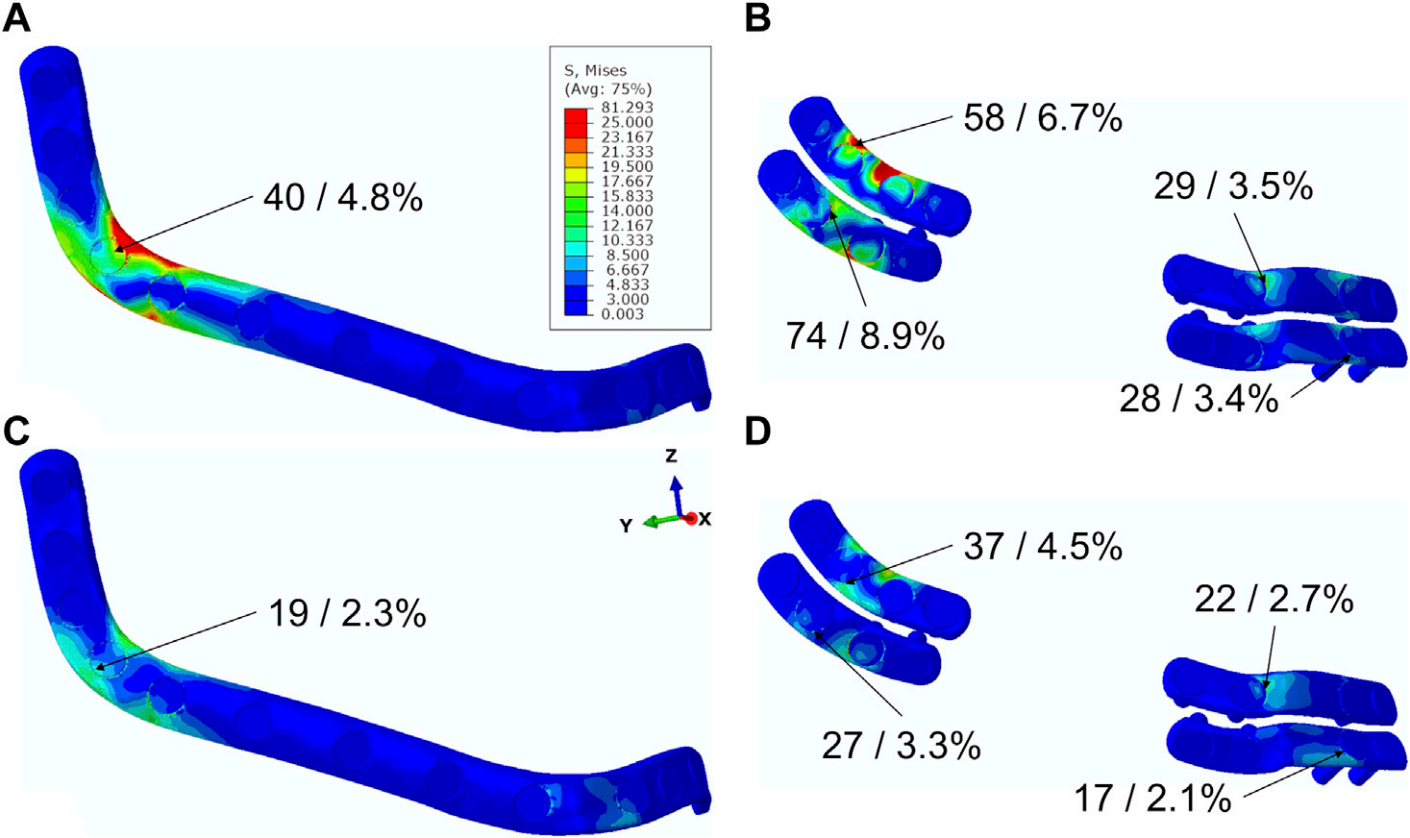
Stress distribution in plates for different plating systems in biting tasks UNI **(A,B)** and INC **(C,D)**, maximum von Mises stress values in MPa, and percentages of maximum stress values in relation to yield stress of Titanium.

For both plating configurations, under both biting conditions, stresses in both plating systems were predicted below the yield strength of the material Ti-6AI-4V (Figure 4). Higher stresses were predicted in the miniplate compared with the reconstruction plate fixation system, in both biting scenarios. The largest stresses were observed in the distal interosseous gap area for both plating systems and biting tasks. Lower peak stresses were determined in both plating systems for INC in comparison with UNI biting (Figure 4).

## 4 Discussion

Due to high rates of osseous non-union after mandible reconstruction with free flaps (Rendenbach et al., 2019; Knitschke et al., 2022), osteosynthesis is a major subject of research in maxillofacial surgery (Brown et al., 2017; Rendenbach et al., 2018; Rendenbach et al., 2019). The requirements of fixation systems are to provide adequate mechanical integrity and to induce beneficial mechanical strains within the healing region to promote bone formation (Rendenbach et al., 2019). If the strains are too low, bone is resorbed due to disuse, whereas too large strains can lead to microdamage and, subsequently, bone resorption (Claes and Heigele, 1999; Lacroix and Prendergast, 2002; Postigo et al., 2014; Ghiasi et al., 2017). Due to their application advantages in comparison to conventional fixation systems, CAD/CAM reconstruction plates are increasingly used in mandibular reconstruction (Tarsitano et al., 2014; Bouchet et al., 2018; Rendenbach et al., 2018; Knitschke et al., 2022). Moreover, CAD/CAM miniplates have been recently proposed as an easily applicable fixation system with potential advantages over reconstruction plates in the postoperative management (Kreutzer et al., 2022a; Kreutzer et al., 2022b). While CAD/CAM reconstruction plates have been found to be more rigid in comparison to conventional fixation systems, biomechanical conditions induced by CAD/CAM miniplates remain unknown (Rendenbach et al., 2017; Steffen et al., 2020; Kreutzer et al., 2022a).

Thus, the aim of this study was to investigate the biomechanics of CAD/CAM manufactured reconstruction plates in comparison to CAD/CAM miniplates in the initial healing phase after mandibular reconstruction using a one segmental fibula free flap. In the distal intersegmental gap, larger strains were found for reconstruction plate fixation, while in the mesial intersegmental gap, larger strains were induced with miniplate fixation. Taking into account the role of mechanical strains on bone healing (Claes and Heigele, 1999), these findings suggest potentially different healing outcomes depending on the fixation in the mesial and distal intersegmental gaps.

In this study, we found average strain values within the interosseous gaps ranging from 0.07% to 0.61%. A previous FE study determined median strains ranging from 0.6% to 2.0% in a mandibular fracture stabilized with 1 mm thick miniplates and a bite force of 100 N in unilateral biting (Orassi et al., 2022). Considering the higher bite force of 100 N in the study by Orassi et al. compared to 45 N in the present study, the strain values found in this study are in line with the existing literature.

Regarding stresses in the fixation systems, a previous study investigated the biomechanics of conventional miniplates and reconstruction plates in a two segmental defect (Park et al., 2018) and found a qualitatively similar stress distribution within the plates as the ones determined in this study for the CAD/CAM plates.

In the postoperative process, 2 weeks after mandibular fracture reposition, bite force has been experimentally found to be in the range of 35–65 N (Gheibollahi et al., 2021), which is in accordance with the findings of the present study. Previous studies investigating fibula free flap using FE models have reported bite forces of 150 N, according to long-term postoperative outcomes described in the literature (Curtis et al., 1997; Atik et al., 2016; Sakuraba et al., 2017; Kucukguven and Akkocaoğlu, 2020). However, to our knowledge, there are no functional measurements of postoperative bite forces in the initial healing phase and over time after fibula free flap reconstruction. Compared to experimentally found long-term bite force values after mandibular reconstruction with a reconstruction plate and unilateral biting, this model predicted 25% of the bite force measured in previous studies (Sakuraba et al., 2017). Due to factors like postoperative pain, swelling, and intraoperative muscle detachment we consider this ratio adequate. The model presented here is based on a previous study (Orassi et al., 2021), where computer model predictions were compared to the existing literature.

In the literature, reconstruction plates are characterized as rigid due to their large geometry and bicortical fixation (Steffen et al., 2020; AO Surgery Reference, 2021a). In contrast, miniplates are known to be load-sharing and less rigid due to their smaller geometry and fixation with monocortical screws (Champy et al., 1978; AO Surgery Reference, 2021a). Interestingly, in the distal healing site, reconstruction plate fixation resulted in higher intersegmental strains compared to miniplate fixation. In this study, monocortical fixation using screws of 7 mm in length could not be achieved on the mandibular angle due to a very thin bone in this region. Therefore, the load-sharing concept of miniplates was not fully applicable to the miniplates in the distal healing region. Anatomically very thin bone in the angular region has been described in a previous study (Ozdemir et al., 2013).

Mechanical strains have been previously shown to play a key role in bone regeneration (Claes and Heigele, 1999; Yoda et al., 2018). In this study, we found higher strain values in the mesial healing site with miniplate fixation and in the distal healing site with reconstruction plate fixation. Assuming a positive correlation between mechanical strains and bone formation on the mandible (Yoda et al., 2018; Orassi et al., 2022), the use of monocortically fixed, load-sharing miniplates in the mesial region could be beneficial to the healing process. Recently, higher rates of pseudarthrosis have been reported in the mesial compared with the distal interosseous gap when using CAD/CAM reconstruction plate fixation (Steffen et al., 2022), which coincides with the lower levels of mechanical strains in the mesial side determined for reconstruction plates in this study. These findings clinically support our assumption that reconstruction plates might be biomechanically beneficial for bone healing in the distal healing site whereas miniplates could improve the bone healing outcome in the mesial healing site. Furthermore, recent studies stated CAD/CAM miniplates as possible solutions to intraoperative and dental rehabilitation problems, especially in the mesial areas (Kreutzer et al., 2022a; Kreutzer et al., 2022b). Therefore, in the mesial healing site, the use of miniplates could be beneficial from a surgical and biomechanical point of view. A fixation with one reconstruction plate in the distal healing site and two miniplates in the mesial healing site, as proposed by Kreutzer et al. (2022a) could combine positive biomechanical effects on bone healing on both healing sites.

In this study, we did not predict fixation failure in any of the configurations investigated, where the highest ratio of maximum stress to yield stress was found to be 8.9%. However, the peak stress in the distal miniplates was found to be over 75% (for UNI) and 40% (INC) higher than in the distal areas of the reconstruction plates. Thus, CAD/CAM reconstruction plates seem to have higher mechanical integrity compared to CAD/CAM miniplates. This is in accordance with previous studies that found small failure rates in titanium CAD/CAM systems, especially reconstruction plates (Steffen et al., 2020; Kreutzer et al., 2022a). Alternative fixation materials with much smaller yield stresses than titanium have been proposed in maxillofacial surgery (Prasadh et al., 2020; Orassi et al., 2022). Since the distal miniplates had an over 70% higher (for INC) and 150% higher (for UNI) peak stress than the mesial miniplates, the use of alternative materials with smaller yield stress could be primarily indicated in the mesial areas regarding their mechanical integrity.

This study presents several limitations that need to be mentioned. We only investigated one patient that was reconstructed using CAD/CAM plates. Future research is needed to investigate the influence of patient-specific properties, like anatomical differences. Moreover, even though maxillofacial surgeons tend to prefer CAD/CAM plates, those might not be the standard in simple one segmental reconstruction due to their excessive costs (Rendenbach et al., 2018). However, recent studies showed the use of patient-specific plates also in one segmental defects and revealed an increase in precision and functional outcome when using CAD/CAM plates (Kreutzer et al., 2022a; Kreutzer et al., 2022b; Knitschke et al., 2022; Steffen et al., 2022). In future studies, the developed methodology can be extended to investigate more complex (multi-segmental) reconstruction cases and different fixation systems, e.g., compare patient-specific plates to conventional plates. In addition, assumptions regarding muscle forces were made to simulate a postoperative scenario. These assumptions were guided by clinical experience, however quantitative results on post-operative muscle forces in the initial healing phase are not available. We determined bite forces of 45 N in unilateral biting, which are in agreement with reported measurements of patients with reduced chewing activity requiring soft food intake (Gibbs et al., 1981; Gheibollahi et al., 2021). Simplified screws and tie constraints were applied to minimize computational costs in locking screw simulation. Since we were not interested in the local stresses around the screws, this can be considered a suitable assumption, however more detailed modeling would be needed to investigate the biomechanics surrounding specific screws. Lastly, homogeneous material properties were assumed for the bony tissue although bone is known to be a heterogeneous material (Blanchard et al., 2013). However, within the present study, the bony tissue was not evaluated as a ROI, but the interosseous gap. This region has much softer material properties than the bone itself and therefore the material properties of the bone do not affect the predicted strains in the intersegmental gaps.

In conclusion, mechanical strains in the reconstructed mandible vary depending on the healing site, biting task, and fixation system. Concordant to clinical findings regarding surgery procedure, CAD/CAM miniplates in the distal healing region do not seem beneficial biomechanically, whereas, in the mesial healing region, they could be a strain-inducing, stable alternative to established fixation systems. Further studies are needed to determine the ranges of mechanical strains promoting bone regeneration in the mandible for further optimization of fixation systems.

## Data Availability

The datasets presented in this article are not readily available because they present some patented elements. Requests to access the datasets should be directed to SC, sara.checa@charite.de.
